# An uncommon electrocardiogram presentation of bigeminy: ECG Challenge

**DOI:** 10.1093/ehjcr/ytae392

**Published:** 2024-08-01

**Authors:** Avinash Jeewooth, Atul Kaushik, Aparna Jaswal

**Affiliations:** Department of Electrophysiology, Fortis Escorts Heart Institute (FEHI), Okhla, New Delhi 110025, India; Department of Cardiology, Fortis Escorts Heart Institute (FEHI), Okhla, New Delhi 110025, India; Department of Electrophysiology, Fortis Escorts Heart Institute (FEHI), Okhla, New Delhi 110025, India

A 52-year-old male with a past history of hypertension, presented with pre-syncope for 15 days. His electrocardiogram is shown in *Figure 1*. His 2D echocardiogram (ECG), Cardiac-MRI, and coronary angiography were normal.

**Figure 1:**
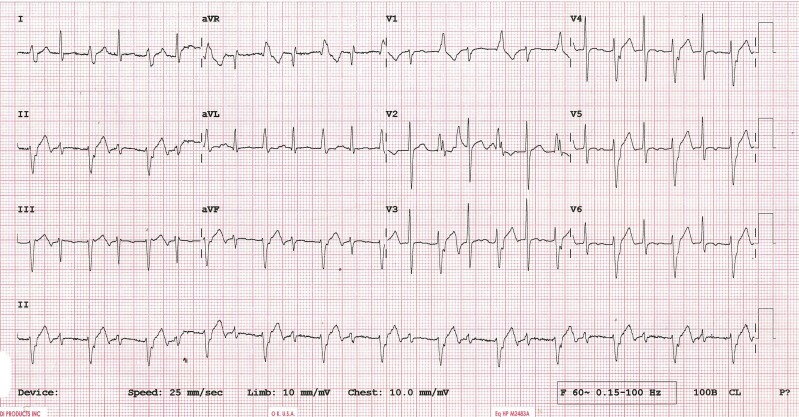
12-lead echocardiogram (ECG) of the patient.

##  

### Question 1

The ECG shows an alternating pattern of narrow QRS complex with wider QRS complex. What is the likely diagnosis?

- Ventricular bigeminy.
- Atrial bigeminy with aberrancy.
- Ventricular tachycardia with capture beats.
- Ventricular tachycardia with fusion beats.
- SVT with aberrancy.

Answer: C.

#### Discussion and explanation

The heart rate in the ECG is around 120 b.p.m.

The wider QRS complex has a right bundle branch block pattern with a superior axis. The QRS duration of the complex is around 120 msec. The RS interval in the precordial leads is short. The broad complexes were not preceded by P waves. These findings favour a ventricular tachycardia (VT) originating close to the conduction system. The diagnosis is left posterior fascicular VT with alternating capture beats.

The narrower QRS complex is a capture beat. These beats are more commonly seen when the tachycardia rate is slow. The rate of the VT was unchanged.

Kindly refer to Supplementary material online, *Image* S*1*, for details.

### Question 2

Which pharmacological drug is most effective against idiopathic fascicular VT?

- Lidocaine.
- Esmolol.
- Amiodarone.
- Verapamil.
- Adenosine.

Answer: D.

#### Discussion and explanation

Verapamil is the first-line treatment for acute termination of fascicular VT (ESC Guidelines Class IC recommendation).^1^ Fascicular VT is a macro-reentrant tachycardia which incorporates LPF as one limb of the circuit and abnormal Purkinje tissues as the other limb. These abnormal Purkinje tissues are calcium-dependent and hence the name ‘verapamil-sensitive idiopathic LV-VT’.

### Question 3

What is the best chronic management option for this patient?

- Catheter ablation.
- Observation.
- Medical therapy.
- No follow-up required.
- Coronary angioplasty.

Answer: A.

#### Discussion and explanation

Catheter ablation is highly effective with a reported success rate of >85%.^2^

Kindly refer to Supplementary material online, *Image S2*, for details.

## Supplementary Material

ytae392_Supplementary_Data

## Data Availability

The data that support the findings of this study are available in the Supplementary material. Any other supporting data will be available from authors upon request.

## Supplementary material

**Consent:** Written consent was obtained from the patient for this manuscript.

**Funding:** No funding was required for this study.
