# Supplementary material for: An uncommon electrocardiogram presentation of bigeminy: ECG Challenge
Source: Eur Heart J Case Rep. 2024 Aug 1;8(8):ytae392. doi: 10.1093/ehjcr/ytae392 (PMC11328535; doi:10.1093/ehjcr/ytae392)
Supplement: ytae392_Supplementary_Data [file ytae392_supplementary_data.docx]

**Supplemental Material**


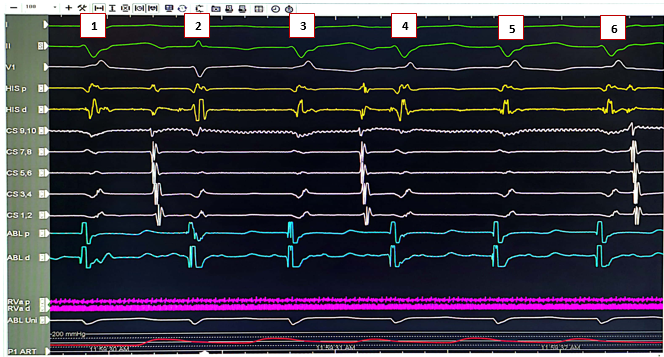


The **supplementary image 1** shows the electrograms of fascicular VT during electrophysiogical study. There is AV dissociation with ventricular rate higher than sinus rate. Three different morphologies of QRS complexes can be observed in lead V1. The QRS complexes 1, 3, 5 and 6 have the same morphology and they likely represent the fascicular VT with retrograde His activation as seen on the His EGM. The QRS 2 is similar to the sinus beat as shown in the above ECG with normal AH and HV interval and this QRS complex is a capture beat. The slowing of the tachycardia might have led to emergence of capture beat. The QRS 4 is a fusion beat with a normal AH and negative anterograde HV interval. Note that the His bundle is not a component of the VT circuit.


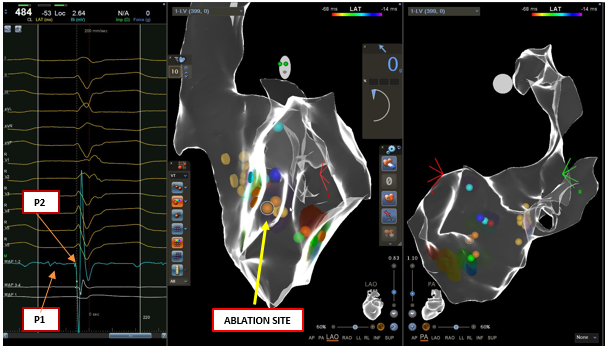


The **supplementary image 2** shows the target ablation site which is the region of left posterior fascicle localized in the inferoposterior LV septum. The LV was mapped using the 3D CARTO electroanatomic mapping system. The site of earliest ventricular activation was targeted during VT. Purkinje potential (P2) can be seen preceding the ventricular activation. A late diastolic potential (P1 or pre-Purkinje potential) can also be appreciated which precedes the P2. Radiofrequency lesions over this site successfully terminated the VT.
